# Supplementary material for: Association between plaque vulnerability and neutrophil extracellular traps (NETs) levels: The Plaque At RISK study
Source: PLoS One. 2022 Jun 9;17(6):e0269805. doi: 10.1371/journal.pone.0269805 (PMC9182254; doi:10.1371/journal.pone.0269805)
Supplement: S1 Fig — (A) Histograms of MPO-DNA complex and histone-DNA levels. (B) Median levels with interquartile range of MPO-DNA complex and histone-DNA for groups with low and high levels. To be able to use MPO-DNA or histone-DNA levels as dependent variable in the logistic regression models, the cut-off value was set at 50% of the values, resulting in a cut-off of 23 mAU for MPO-DNA and 69 mAU for histone-DNA. mAU, milli-arbitrary units; MPO, myeloperoxidase. (DOCX) [file pone.0269805.s001.docx]

**
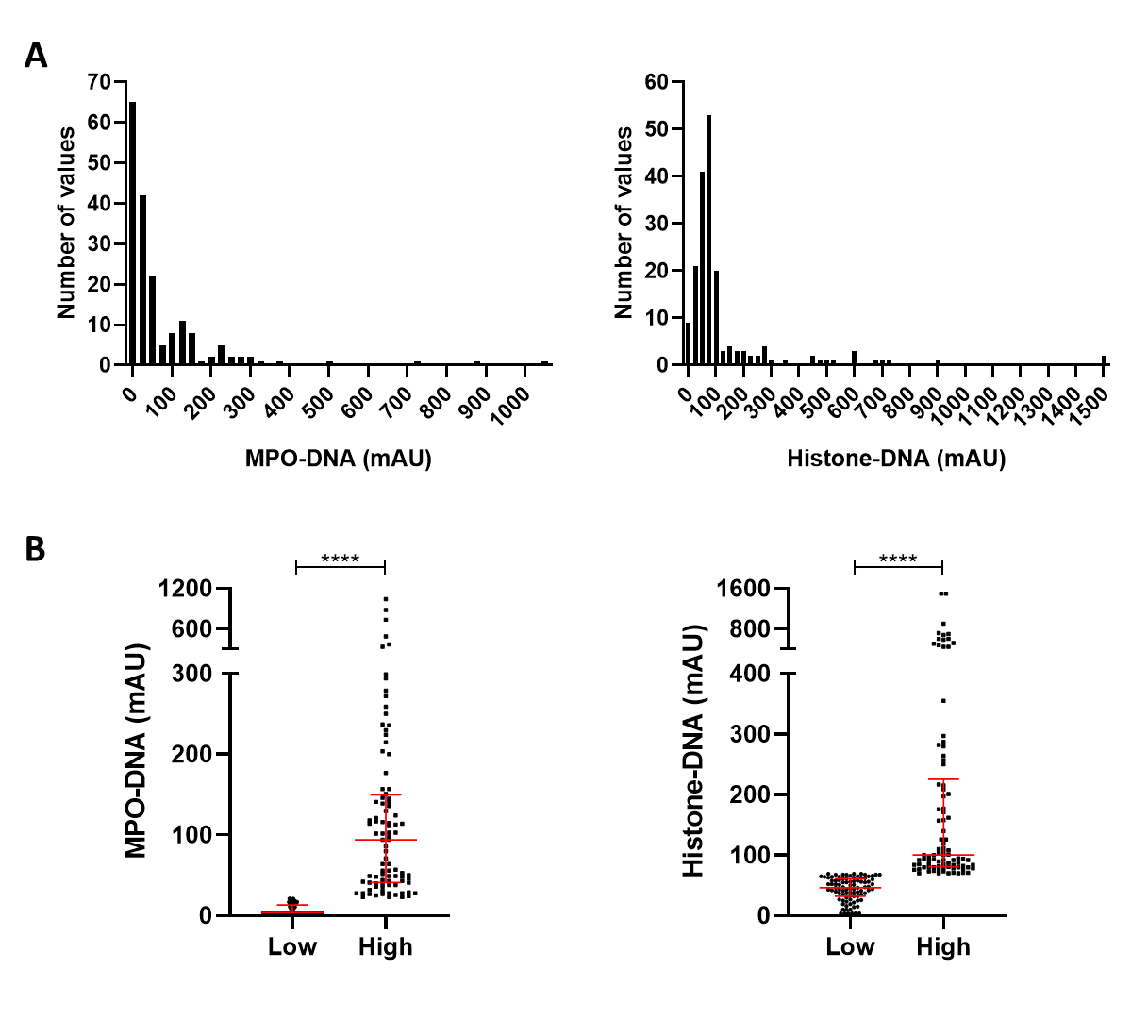
**

**S1 Figure. Distribution of MPO-DNA complex and histone-DNA levels and classification into two groups**
(A) Histograms of MPO-DNA complex and histone-DNA levels. (B) Median levels with interquartile range of MPO-DNA complex and histone-DNA for groups with low and high levels. To be able to use MPO-DNA or histone-DNA levels as dependent variable in the logistic regression models, the cut-off value was set at 50% of the values, resulting in a cut-off of 23 mAU for MPO-DNA and 69 mAU for histone-DNA. mAU, milli arbitrary units; MPO, myeloperoxidase.
