# Supplementary material for: Association between plaque vulnerability and neutrophil extracellular traps (NETs) levels: The Plaque At RISK study
Source: PLoS One. 2022 Jun 9;17(6):e0269805. doi: 10.1371/journal.pone.0269805 (PMC9182254; doi:10.1371/journal.pone.0269805)
Supplement: S2 Fig — The different vulnerable plaque components were combined into components, using the varimax rotation method. Component 1 has the highest eigenvalue and was therefore used as ‘vulnerability index’ in this study. (DOCX) [file pone.0269805.s002.docx]

**
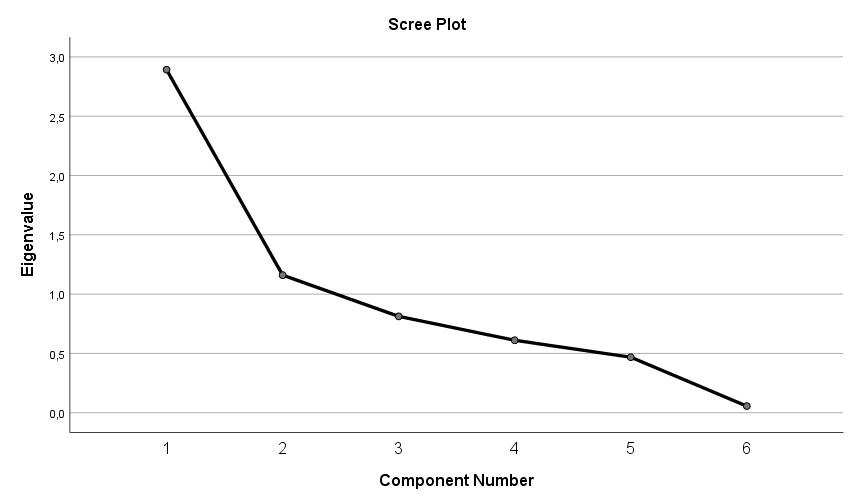
**

**S2 Figure.** **Scree plot for the principal component analysis as presented in Table 3.**

The different vulnerable plaque characteristics were combined into components, using the varimax rotation method. Component 1 has the highest eigenvalue and was therefore used as ‘vulnerability index’ in this study.
