# Supplementary material for: Association between plaque vulnerability and neutrophil extracellular traps (NETs) levels: The Plaque At RISK study
Source: PLoS One. 2022 Jun 9;17(6):e0269805. doi: 10.1371/journal.pone.0269805 (PMC9182254; doi:10.1371/journal.pone.0269805)
Supplement: S1 Table — Data is presented as mean (SD) for normally distributed variables, median [25th-75th percentile] and number (percentage) for frequencies. P-value represents the difference between patients with and without use of statins or antithrombotic medication tested by the independent students’ t-test, Mann-Whitney U test or Chi-square test. *indicates p-value below 0.05. Abbreviations: BMI, body mass index; mAU, milli-arbitrary units; MPO, myeloperoxidase; NETs, neutrophil extracellular traps; TIA, transient ischemic attack. (DOCX) [file pone.0269805.s003.docx]

**S1 Table.** **Clinical characteristics in the subgroups of patients stratified by statin and antithrombotic medication use prior to the index event**

| **Clinical characteristics** |  | **Patients without medication (n=72)** | **Patients with medication (n=109)** |  | **P-value** |
| --- | --- | --- | --- | --- | --- |
| Age (years) |  | 64 ± 10 | 69 ± 8 | **<** | **0.01*** |
| Sex (male) |  | 46 (64%) | 88 (81%) |  | **0.01*** |
| BMI |  | 26 ± 4 | 27 ± 5 |  | 0.05 |
| Classification event |  |  |  |  | 0.67 |
| TIA |  | 33 (46%) | 43 (39%) |  |  |
| Stroke |  | 31 (43%) | 51 (47%) |  |  |
| Amaurosis fugax |  | 8 (11%) | 15 (14%) |  |  |
| Diabetes mellitus |  | 9 (13%) | 35 (33%) | **<** | **0.01*** |
| Hypercholesterolemia |  | 39 (59%) | 103 (95%) | **<** | **0.01*** |
| Hypertension |  | 38 (54%) | 90 (83%) | **<** | **0.01*** |
| History of cardiovascular disease |  | 12 (17%) | 77 (71%) | **<** | **0.01*** |
| Current smoking |  | 20 (29%) | 19 (18%) |  | 0.09 |
| Medication use prior to event |  |  |  |  |  |
| Statins |  |  | (100%) |  |  |
| Antihypertensive drugs |  | 24 (33%) | 86 (79%) | **<** | **0.01*** |
| Antidiabetic drugs |  | 4 (6%) | 29 (27%) | **<** | **0.01*** |
| Antiplatelet drugs |  |  | (100%) |  |  |
| Anticoagulants |  |  | (100%) |  |  |
| NETs levels (MPO-DNA) (mAU) |  | 26 [4-111] | 23 [4-94] |  | 0.75 |

Data is presented as mean (SD) for normally distributed variables, median [25^th^-75^th^ percentile] and number (percentage) for frequencies. P-value represents the difference between patients with and without prior use of statins or antithrombotic medication tested by the independent students’ t-test, Mann-Whitney U test or Chi-square test. *indicates p-value below 0.05. Abbreviations: BMI, body mass index; mAU, milli-arbitrary units; MPO, myeloperoxidase; NETs, neutrophil extracellular traps; TIA, transient ischemic attack.
