# Supplementary material for: Association between plaque vulnerability and neutrophil extracellular traps (NETs) levels: The Plaque At RISK study
Source: PLoS One. 2022 Jun 9;17(6):e0269805. doi: 10.1371/journal.pone.0269805 (PMC9182254; doi:10.1371/journal.pone.0269805)
Supplement: S2 Table — The different vulnerable plaque characteristics were combined into one component representing the vulnerability of plaques using principal component analysis, while stratified in two groups based on the use of statin and antithrombotic medication prior to the index event. Similar components were generated in both groups, which were also comparable to the component generated in the total population (Table 3). Abbreviations: IPH, intraplaque hemorrhage; LRNC, lipid-rich necrotic core; PCA, principal component analysis. Bold values represent highest factor loadings per component. (DOCX) [file pone.0269805.s004.docx]

**S2 Table.** **Varimax Rotated Component Matrix derived from PCA for the subgroups of patients stratified by statin and antithrombotic medication use prior to the index event**

|  | **Patients without medication (n=72)** | **Patients with medication (n=109)** |
| --- | --- | --- |
| Relative LRNC volume (%) | **0.928** | **0.943** |
| Relative IPH volume (%) | **0.894** | **0.923** |
| Thin or ruptured fibrous cap | **0.739** | **0.761** |
| Ulceration size | **0.477** | **0.453** |
| Relative calcification volume (%) | -0.202 | 0.048 |
| Plaque volume (mm^3^) | **0.570** | **0.619** |
| *Eigenvalue* | 2.808 | 2.912 |
| *Variance explained (%)* | 46.7 | 48.5 |

The different vulnerable plaque characteristics were combined into one component representing the vulnerability of plaques using principal component analysis, while stratified in two groups based on the use of statin and antithrombotic medication prior to the index event. Similar components were generated in both groups, which were also comparable to the component generated in the total population (Table 3). Abbreviations: IPH, intraplaque hemorrhage; LRNC, lipid-rich necrotic core; PCA, principal component analysis. Bold values represent highest factor loadings per component.
