# Supplementary material for: Association between plaque vulnerability and neutrophil extracellular traps (NETs) levels: The Plaque At RISK study
Source: PLoS One. 2022 Jun 9;17(6):e0269805. doi: 10.1371/journal.pone.0269805 (PMC9182254; doi:10.1371/journal.pone.0269805)
Supplement: S3 Table — Logistic regression with two categories of MPO-DNA as dependent variable (high vs low) and plaque characteristics as independent variables, adjusted for age, sex and time between index event and blood sampling. OR for plaque volumes is presented for 1000 mm3. *indicates p-value below 0.05. CI, confidence interval; IPH, intraplaque hemorrhage; LRNC, lipid-rich necrotic core; OR, odds ratio. (DOCX) [file pone.0269805.s005.docx]

**S3 Table. Association between plaque characteristics and MPO-DNA levels in subgroups stratified by statin and antithrombotic medication use prior to the index event**

|  | **Patients without medication (n=72)** | | **Patients with medication (n=109)** | |
| --- | --- | --- | --- | --- |
| **Plaque characteristic** | **OR [95% CI]** | ***p*-value** | **OR [95% CI]** | ***p*-value** |
| IPH presence | 5.29 [1.54-18.06] | **0.01*** | 0.72 [0.30-1.71] | 0.46 |
| Relative IPH volume (%) | 1.19 [1.04-1.35] | **0.01*** | 1.00 [0.94-1.06] | 0.91 |
| LRNC presence | 2.45 [0.78-7.69] | 0.13 | 0.61 [0.26-1.47] | 0.27 |
| Relative LRNC volume (%) | 1.12 [1.03-1.21] | **0.01*** | 0.98 [0.94-1.02] | 0.36 |
| Ulceration presence | 5.93 [1.38-25.37] | **0.02*** | 2.04 [0.81-5.13] | 0.13 |
| Ulceration size | 1.70 [0.97-3.00] | 0.07 | 1.14 [0.77-1.69] | 0.52 |
| Thin or ruptured fibrous cap | 1.51 [0.55-4.13] | 0.42 | 0.75 [0.31-1.80] | 0.51 |
| Calcification presence | 0.85 [0.18-3.95] | 0.84 | - | 0.99 |
| Relative calcification volume (%) | 1.01 [0.92-1.11] | 0.86 | 1.02 [0.95-1.10] | 0.59 |
| Plaque volume (mm^3^) | 0.79 [0.42-1.48] | 0.46 | 3.90 [1.16-13.13] | **0.03*** |

Logistic regression with two categories of MPO-DNA as dependent variable (high vs low) and plaque characteristics as independent variables, adjusted for age, sex and time between index event and blood sampling. OR for plaque volumes is presented for 1000 mm^3^. *indicates *p*-value below 0.05. CI, confidence interval; IPH, intraplaque hemorrhage; LRNC, lipid-rich necrotic core; OR, odds ratio.
